# Notes on the Manufacture and Cost of Charcoal for Sanitary Purposes

**Published:** 1855-09

**Authors:** E. H. Durden


### NOTES ON THE MANUFACTURE AND COST OF CHARCOAL FOR SANITARY PURPOSES.

By E. H. Durden, F.R.S.

*Manufacture of Charcoal in Heaps or Mounds.* This mode of making charcoal, which has been known for 2,000 years, is in use in most parts of the country. When made from wood, a conical heap or mound is carefully built up, and covered with sods of earth. The pile is kindled by means of ignited wood, thrown into the hollow space left in the centre of the mound. In this mode of making charcoal, the expense of conveying the wood to any given locality is saved, the charcoal being made on the spot where the wood is felled. The price of wood and of labour differs so much in various localities, that it is difficult to give an idea of the average cost of charcoal made in this way. Taking the value of wood on the spot at 6*s*. per ton, in a tolerably dry state, the cost of such charcoal would, with the addition of labour, be about 33*s*. per ton *on the spot.* Add to this carriage to a given locality, and expense of grinding, the *actual cost* of the ground charcoal may be estimated at 45*s*. per ton.

Various methods have been tried for collecting the pyrolignous acid, etc., formed in the process of carbonisation; but it has been found that collecting these products from the mound generally does more damage than it is worth. The charcoal obtained by the above process answers better than any other for metallurgical purposes; but it is doubtful whether it is so well adapted for sanitary purposes as the cylinder-made charcoal.

*Peat* may also be carbonised in heaps or mounds, in the same way as wood charcoal; although, from the great contraction which peat undergoes when carbonised, difficulties are experienced which do not occur in the case of wood. This mode of carbonisation is adopted in France, Bavaria, Saxony, and Bohemia; and the cost of peat charcoal produced is estimated at from 14s. to 16*s*. 6*d*. per ton. I have no data as to the probable cost in this country; but, as the quantity of charcoal obtained in Bavaria is equal to 25 per cent. of the weight of the peat employed, I have no doubt it could be manufactured in Ireland at a price equally low.

*Manufacture of Charcoal in Iron Cylinders.* This is the mode adopted in the preparation of wood spirit and acetic acid. Large iron retorts, set in brickwork, are filled with wood, and heated externally. Water, wood spirit, acetic acid, tar, etc., pass off in the form of vapour, which is condensed by suitable means, and these various products separated by subsequent distillation. Incondensible gases are also given off. The charcoal, which remains behind in the retorts, is drawn out into iron boxes and cooled. The average annual product, in a large establishment, would not exceed 23 per cent. In this mode of manufacturing charcoal, the cost of the wood is a more expensive item than in the preceding mode, inasmuch as the carriage from the place where it was felled to the works where it is carbonised has to be added to the original cost. The cost of wood delivered at the works varies considerably in different parts of the kingdom; but, taking an average value, and adding thereto the expenses of fuel and labour, and wear and tear of apparatus, the cost of charcoal made in this way would be upwards of £5 per ton. It answers the purpose of the manufacturer of gunpowder to make charcoal in this way, without troubling himself to collect any of the other products, because the charcoal is the most important ingredient in the gunpowder, and the expense of obtaining it, suitable for the purpose required, is compatively of little importance; but it will not pay to make disinfecting charcoal by this process. The acetic acid and wood spirit obtained, at present enable the manufacturer of cylinder charcoal to carry on his works; but it would not do to multiply such establishments as these for the sake of the charcoal only, as the increased supply of wood spirit—an article at present of limited consumption, and likely under the operation of the Spirit of Wine Act of the present session to be still more limited—would so much exceed the quantity required for consumption, that the consequent reduction in price necessary to effect sales would speedily close the manufactory. Granulated cylinder charcoal, suitable for sanitary purposes, may be obtained at 35*s.* to 40*s.* per ton at the works.

This mode of carbonising peat by the iron cylinder has been tried at Dartmoor, in Devonshire, on a large scale. The experiment, however, was found to be not sufficiently remunerative, and was therefore abandoned. The peat is so light of weight, that a very considerable number of retorts are required for the carbonisation of such a quantity of material as would be worth while to operate on daily: hence a very great outlay in plant is requisite; the labour of charging and discharging is also proportionably great. Much longer time is required for the carbonisation of peat than of wood, and the relative products obtained are not so large in quantity. The commercial working of this process gave the following results:—

| | |
|---|---|
| Peat employed to furnish the charcoal | 100 tons. |
| Peat required for fuel . . . | 100 |
| | 200 at 2*s.* 6*d.* per ton. |

Products obtained:—Charcoal, 33 tons; peatine, 100 gallons; heavy oil, 50 gallons; pyroxylic spirit, 30 gallons; acetic acid, 168 gallons; crude tar of the consistence of tallow, 33 cwt. 0 qrs. 14 lbs.; sulphate of ammonia, 7 cwt. 2 qrs. 0 lbs.; peat ashes, 8 tons.

*Manufacture of Charcoal from Sawdust, Spent Dye Woods, etc.* Sawdust, spent dye woods, spent bark from the tan pits, etc., are all capable of yielding, by destructive distillation, wood spirit, and acetic acid. The carbonisation of these materials was found not to answer in a commercial point of view, on a large scale, whilst using the ordinary iron cylinders; for the sawdust, in close contact with the heated cylinder, becoming carbonised, forms a layer of non-conducting material, presenting a complete barrier to the passage of the heat to the interior of the mass. To obviate this difficulty, the late Mr. Halliday, of Salford, devised and patented the following plan. The raw material is introduced into a hopper, placed above the front end of an ordinary iron cylinder, about fourteen inches in diameter. In this hopper a vertical screw revolves, conveying the material, and in the proper quantities, to the cylinder, which is placed in a horizontal position, and heated by means of a furnace in the usual manner. Another revolving screw or worm keeps the material in the retort in constant agitation, and at the same time moves it forward to the end. During its progress through the cylinder, the material is completely carbonised, and all the volatile products disengaged. At the extreme end of the cylinder two pipes branch off; one passing downwards and dipping into a cistern of water, or an air-tight iron vessel, into which the charcoal falls; the other pipe, passing upwards, conveys the vapours and gases into the condensing apparatus. In Muspratt's *Dictionary of Chemistry*, article "Acetic Acid", a comparison is given of the quantities of some of the products obtained by this process, as compared with the ordinary cylinder plan. The produce of charcoal of the screw process is not given; but, assuming it to be about 600 lbs. from a ton of sawdust, and putting the cost of the sawdust at 5*s*. per ton, the cost of such charcoal, including labour, fuel, and other expenses, would be about 40*s*. per ton. The density of the pyrolignous acid produced is stated to be 10° Twaddle (specific gravity 1,050), whilst the density of that obtained by ordinary cylinders is but 6° Twaddle (specific gravity 1,030). We are inclined to believe that this increased density is due to the presence of resinous and other matters, which render a fair comparison obtainable only by comparing the saturating power of the two samples. As the quantity of wood spirit is not given, and as the product in real acid is, we think, erroneously given, we are unable to make a calculation of the actual cost of the charcoal to the manufacturer. It is sold at 40*s*. per ton.

*Manufacture of Charcoal by Sulphuric Acid.* This process is the carrying out, on a commercial scale, of the well known lecture experiment, shewing the composition of woody fibre. A patent has been taken out for this mode of manufacturing charcoal by Mr. Longmaid, who estimates the cost of production at the low price of 12*s*. per ton. The sawdust is first steeped in dilute sulphuric acid until thoroughly saturated, after which it is exposed, on a kiln or suitable apparatus, to a temperature of about 250° Fahr., when it is found to be completely carbonised. In this case, the product of charcoal is much greater than in either of the other modes, which may be thus accounted for: In the carbonisation of wood, whether in mounds or cylinders, a variety of products are formed, of which carbon forms a constituent. A certain quantity of the carbon originally contained in the wood is therefore consumed in the formation of these products; but in the preparation of sulphuric acid charcoal, only the oxygen and hydrogen of the wood are removed, leaving the whole of the carbon in the state of charcoal. Mr. Longmaid estimates the product of charcoal at, I believe, about 55 per cent. It becomes a question how far this charcoal would answer as a deodoriser. I have not made any experiment on the subject; but I know that Mr. Longmaid looks to this use of it as furnishing a market for its sale.

*Manufacture of Peat Charcoal in Furnaces, without using Fuel* (*Mr. Jasper Rogers' patent*). Immediately the blocks are cut from the bog, they are placed in exposed situations, so that the air may circulate freely around and through them, and assist to drive off the moisture from the peat. Thus, in the course of a short time, according to the state of the weather, it will have become sufficiently dry for conveyance to the drying house, where the remaining moisture is driven off by stacking it against an air-chamber, heated by the surplus heat from the carbonising furnaces.

The carbonising furnaces are of wrought iron, pyramidically shaped, the base measuring about five feet: they run on wheels, and are filled through an opening on the top: the peat is ignited by the introduction of some burning peat. In the course of a few hours the peat becomes perfectly charred; and the damper attached to the furnace being closed, the charcoal is allowed to cool.

The following estimate of the cost of the production of peat charcoal by this process was supplied by the engineer of the Irish Amelioration Society, Mr. Yarrow:—

| | £ | *s.* | *d.* |
|---|---|---|---|
| Cost of labour in converting 36,000 tons of peat into charcoal . . . . | 8,062 | 8 | 0 |
| Other charges attending the manufacture and conveyance to Dublin . . . | 4,711 | 12 | 0 |
| | £12,774 | 0 | 0 |

The quantity of charcoal produced from 36,500 tons of peat is 12,166 tons, and the cost of manufacture, as shewn above, is 21*s.* per ton; the selling price in Dublin being 40*s.* per ton. The cost of the plant required to produce the above quantity, together with the fee simple of the soil, upon which the annual interest has to be calculated, is under £5,000.

The following is an estimate of the working expenses and produce of a small station, based upon actual practice:—

Cost of erection of station, £310.

| | £ | *s.* | *d.* |
|---|---|---|---|
| Cost of 936 tons of dried peat, cut by contract, at 2*s.* per ton . . . . . | 93 | 12 | 0 |
| Labour in carbonising, granulating, and packing | 140 | 8 | 0 |
| Cartage to railway, at 6*s.* per ton . . | 93 | 12 | 0 |
| Wear and tear and sundries 2*s.* per ton . | 31 | 4 | 0 |
| Patentee's royalty . . . . . | 31 | 4 | 0 |
| Cartage per rail to a market, on the average twelve to fifteen miles, at 2*d.* per ton per mile | 39 | 0 | 0 |
| Total expenses . . . | £429 | 0 | 0 |

Product, 312 tons of charcoal, at 27*s.* 6*d.* per ton, or about 19*s.* on the spot.

*Manufacture of Peat Charcoal by means of Super-Heated Steam.* This process has been patented by Mr. Vignolles, C.E. The *modus operandi* is as follows: The peat is placed in large sheet iron vessels, into which steam, heated to a temperature of from 450° to 600° Fahr. (by being passed through a coil of pipe heated to that temperature in a furnace), is introduced. The steam, in passing through the mass of peat, gives out its excess of heat, which is sufficient to effect the conversion of the material into charcoal. The above process is a modification of the invention of my friend M. Violette (formerly Commissary at the French Government Gunpowder Works of Esquerdes, near St. Omer, Pas de Calais) for carbonising wood for the manufacture of gunpowder.

The following estimate (based on the actual cost of an establishment at work at Friesach, in Prussia) is given by Mr. Vignolles, for an apparatus calculated to produce 11,000 tons of marketable charcoal annually, by means of the super-heated steam process:—

Boilers, twelve carbonising cylinders, masonry, brickwork, refrigerating waggons, etc., etc., £5,000.

| WORKING EXPENSES. | £ | *s.* | *d.* |
|---|---|---|---|
| Cost and transport of 28,080 tons of peat . | 2,106 | 0 | 0 |
| Fuel, labour, interest, repairs, management, etc. | 2,545 | 12 | 6 |
| | £4,651 | 12 | 6 |

Product, 11,232 tons of charcoal, costing 8*s.* 3*d.* per ton.

The Dumfries, Glasgow, and Carlisle Peat Company, estimate the cost of a ton of peat charcoal as follows:—

| | *s.* | *d.* |
|---|---|---|
| Cutting three tons of peat, the quantity required for one ton of charcoal . . . . | 3 | 0 |
| Drying the same . . . . . | 3 | 0 |
| Carbonising ditto . . . . . | 4 | 6 |
| Peat used in drying and carbonising . . . | 2 | 0 |
| Rent of peat ground, say . . . . . | 0 | 6 |
| Cost on the spot . . . . . | 13 | 0 |
| Carriage to market . . . . . | 3 | 6 |
| | 16 | 6 |

The Great Peat Working Company of Ireland estimate the cost of a ton of peat charcoal as follows:—

| | £ | *s.* | *d.* |
|---|---|---|---|
| Cutting three tons of peat, quantity required to make one ton of charcoal . . . | 0 | 3 | 0 |
| Drying by artificial heat three tons . . | 0 | 3 | 0 |
| Carbonising ditto . . . . . | 0 | 4 | 6 |
| Peat used as fuel in drying and carbonising . | 0 | 2 | 0 |
| Royalty or cost of peat . . . . | 0 | 1 | 6 |
| *Carry forward* . . . . | 0 | 14 | 0 |

| | | | |
|---|---|---|---|
| *Brought forward* . . . . | 0 | 14 | 0 |
| Cost on the spot . . . . . | 0 | 14 | 0 |
| Add carriage to seaport town . . . | 0 | 3 | 0 |
| Freight to an English market . . . | 0 | 12 | 0 |
| | £1 | 9 | 0 |

Peat charcoal made in heaps or mounds appears to be the best for metallurgical uses, and that made in retorts and furnaces for sanitary purposes.

*En resumé*, the price of wood, fuel, and labour, varies so much in different localities, that it is impossible to give more than a rough idea of the actual cost of charcoal. The sulphuric acid plan appears the cheapest mode of obtaining charcoal, provided that a sufficient supply of raw material could at all times be readily obtainable at a low price. Mr. Longmaid proposes to carbonise peat in the same way; in this case, the supply of raw material is inexhaustible, so to speak. Peat charcoal is, however, apt to contain a large quantity of uncarbonized peat. There can be no doubt that the cylinder-made wood charcoal is the best for sanitary purposes. The question of comparative cost will, however, depend on the locality where the charcoal may be required. As regards the metropolis, granulated charcoal may be supplied to the wholesale dealers, at such a price as to enable them to *retail* it at from 4*s.* to 5*s.* per cwt.

*Hemslet, Leeds. Sept.* 1855.

---

